# Low, Intermediate, and High Glutamine Levels Are Progressively Associated with Increased Lymphopenia, a Diminished Inflammatory Response, and Higher Mortality in Internal Medicine Patients with Sepsis

**DOI:** 10.3390/jcm14103313

**Published:** 2025-05-09

**Authors:** Filippo Mearelli, Alessio Nunnari, Federica Chitti, Annalisa Rombini, Alessandra Macor, Donatella Denora, Luca Messana, Marianna Scardino, Ilaria Martini, Giulia Bolzan, Noemi Merlo, Fabio Di Paola, Francesca Spagnol, Chiara Casarsa, Nicola Fiotti, Venera Costantino, Verena Zerbato, Stefano Di Bella, Carlo Tascini, Daniele Orso, Filippo Giorgio Di Girolamo, Gianni Biolo

**Affiliations:** 1 Unit of Internal Medicine, Clinica Medica, Department of Medical Surgical and Health Sciences, University of Trieste, Strada di Fiume 447, 34100 Trieste, Italy; 2 Microbiology Unit, University Hospital (ASUGI), Strada di Fiume n° 447, 34137 Trieste, Italy; venera.costantino@asugi.sanita.fvg.it; 3 Infectious Diseases Unit, Clinical Departement of Medical, Surgical, and Health Sciences, University Hospital (ASUGI), Piazzale dell’Ospedale n° 1, 34129 Trieste, Italystefano932@gmail.com (S.D.B.); 4 Infectious Diseases Unit, University Hospital (ASUFC), Via Pozzuolo n° 330, 33100 Udine, Italy; c.tascini@gmail.com; 5 Department of Anesthesia and Intensive Care, University Hospital (ASUFC), Via Pozzuolo n° 330, 33100 Udine, Italy

**Keywords:** sepsis, glutamine, glutamic acid, 5-oxoproline, phenylalanine/tyrosine, leucine

## Abstract

**Background:** The pathophysiological mechanisms underlying altered plasma glutamine concentrations in sepsis remain poorly understood. Identifying clinical, immunological, and metabolic correlates of glutamine fluctuations is crucial to advancing precision medicine, developing targeted therapies, and improving survival outcomes in septic patients. **Methods:** We enrolled 469 patients with sepsis and assessed inflammatory markers—including body temperature, white blood cell count, and C-reactive protein levels—upon admission to the internal medicine unit. Lymphocyte count and plasma concentrations of glutamine, glutamic acid, 5-oxoproline, phenylalanine, tyrosine, and leucine were measured using gas chromatography–mass spectrometry. Patients were stratified into three groups based on plasma glutamine levels. Mortality was recorded at 30 days and 6 months. **Results:** Low, intermediate, and high glutamine levels were observed in 46% (*n* = 217), 47% (*n* = 218), and 7% (*n* = 34) of patients, respectively. Patients with hyperglutaminemia exhibited significantly lower body temperature, white blood cell and lymphocyte counts, C-reactive protein levels, and glutamic acid-to-5-oxoproline ratio (a surrogate marker of glutathione availability), along with elevated phenylalanine levels, leucine levels, and tyrosine-to-phenylalanine ratio (all *p* < 0.01). Metabolic disruption and mortality increased progressively across glutamine level groups. Kaplan–Meier analysis demonstrated significantly higher mortality in patients with elevated glutamine levels at both 30 days (*log-rank p* = 0.03) and 6 months (*log-rank p* = 0.05). **Conclusions:** At baseline, increasing plasma glutamine levels are associated with progressively deeper lymphopenia, more pronounced metabolic derangement, and higher short- and long-term mortality in patients with sepsis.

## 1. Introduction

Sepsis is a life-threatening condition and a major global health burden, accounting for an estimated 49 million cases and 11 million deaths worldwide each year [1]. Glutamine, the most abundant amino acid in the human body, becomes conditionally essential during critical illness, when its demand increases significantly [2,3,4,5]. It serves as a key energy substrate for rapidly proliferating cells, particularly immune cells, and is a crucial precursor for nucleotide biosynthesis. Glutamine also plays vital roles in glutathione production, renal ammoniagenesis, and muscle glycogen resynthesis [2,3,4,5].

In critically ill patients, the mechanisms underlying abnormal plasma glutamine levels remain unclear. Hypoglutaminemia is frequently observed and has been associated with poor outcomes in multiple studies [2,3,4,5]. Experimental models have shown that glutamine supplementation enhances both humoral and cell-mediated immunity, leading to improved survival [6]. These findings have spurred clinical trials investigating whether glutamine administration can reduce mortality in critically ill populations [7]. However, in recent years, an increasing number of Intensive Care Unit (ICU) patients have presented with elevated plasma glutamine levels [8]. Like those with glutamine deficiency, hyperglutaminemic patients also exhibit increased mortality [8,9]. Consequently, the benefits of glutamine supplementation in unselected critically ill cohorts have remained inconclusive [7]. Current European Society for Clinical Nutrition and Metabolism (ESPEN) guidelines recommend glutamine supplementation only in specific conditions such as trauma and burns [7]. Although some non-infectious acute conditions share features with severe infections, sepsis follows a distinct and complex pathophysiological trajectory [10,11]. It is a heterogeneous syndrome, encompassing patients who display either pronounced hyperinflammation or immune suppression [10,11]. In this context, glutamine supplementation may offer survival benefits to select subgroups of clinically stable patients [6,7]. To date, no clinical studies have established a clear link between plasma glutamine concentrations and the extent of inflammation, lymphopenia, metabolic alterations, or prognosis in patients with sepsis admitted to internal medicine units.

Our primary aim was to investigate the relationship between low, intermediate, and high plasma glutamine levels and immune–inflammatory profiles, metabolic abnormalities, and clinical outcomes in patients with sepsis. This study was conducted in the internal medicine department of the University Hospital of Trieste, Italy.

## 2. Materials and Methods

Consecutive adult patients with suspected community-acquired sepsis at Emergency Department (ED) admission who were transferred to the Clinica Medica (internal medicine unit [IMU]) at the University Hospital of Trieste, Italy, between 1 January 2017 and 1 June 2018 were eligible for inclusion. The collection of blood cultures and initiation of empirical antibacterial therapy in patients with a sequential organ failure assessment (SOFA) score of ≥2 served as a surrogate for the suspicion of sepsis at ED admission. Exclusion criteria were age < 18 years and pregnancy. Clinical characteristics at IMU admission were recorded in a dedicated case report form for each patient with suspected sepsis. We focused on clinical data that might reflect the inflammatory response (body temperature, white blood cell count, and serum levels of C-reactive protein) and immune status (lymphocyte count [11,12]) at IMU admission. Patients with chronic immunodeficiency included those diagnosed with Human Immunodeficiency Virus, active lymphoma, leukemia, solid tumors, and individuals undergoing long-term therapy with corticosteroids and/or immunosuppressive agents. Acute liver failure was diagnosed by an International Normalized Ratio (INR) ≥ 1.5 and signs of hepatic encephalopathy. A supratherapeutic INR associated with signs of hepatic encephalopathy was the criterion for acute liver failure in patients receiving vitamin K antagonists. At the end of clinical follow-up, patients classified as infected by the data review committee were included in the final analysis. The nature of the acute illness, its severity, etiology, and source of infection were adjudicated according to definitions detailed in the Supplementary File S1. Regarding the outcome of septic patients, mortality at 30 days and 6 months from IMU admission was recorded.

A blood sample for amino acid assay was drawn at baseline (within 6 h of IMU admission) in all patients with suspected sepsis. Coagulated plasma EDTA was frozen (−80 °C) for subsequent analysis. Levels of amino acids were determined in defrosted plasma samples of patients who were judged to be infected by the data review committee (Supplementary File S1). In order to explore several pathways of metabolic derangements in patients with sepsis, beyond glutamine, we assayed plasma levels of glutamic acid, 5-oxoproline, phenylalanine, tyrosine, and leucine by gas chromatography–mass spectrometry using stable isotopes of amino acids as internal standards [13]. These serve as surrogates of the extent of oxidative stress (glutamic acid and 5-oxoproline [13,14,15]; phenylalanine and tyrosine [16,17,18,19]), glutathione stores (glutamic acid and 5-oxoproline [12,13,14]), stress hormone release (phenylalanine and tyrosine [16,17,18,19]), anabolic resistance, and endogenous release of energy (leucine [18,19,20,21]).

The study was approved by the Ethics Committee of the University of Trieste (Report n°39). The study was conducted in accordance with the Declaration of Helsinki. Each participant or legally authorized next of kin provided informed consent prior to data collection.

### Statistical Analysis

For descriptive statistics, categorical data were reported as absolute frequencies and percentages, while continuous data were presented as medians with interquartile ranges. Clinical variables (including the plasma levels of other amino acids measured in this study) associated with 6-month mortality and an unadjusted *p*-value of ≤0.1 (using the χ^2^ test or Fisher’s exact test) were included in the logistic regression analysis. Odds Ratios (ORs) and 95% confidence intervals (CIs) were calculated for clinical variables that were identified as independent predictors of 6-month mortality. We compared patient characteristics based on glutamine level cut-offs (using Student’s *t*-test, the Mann–Whitney U-test, or Fisher’s exact test, as appropriate), with low, intermediate, and high glutamine levels defined as <400 μmol/L [7], 400–700 μmol/L, and >700 μmol/L [4,9], respectively. The choice of the latter threshold was based on previous studies indicating that glutamine levels above 700 μmol/L are associated with increased mortality [4,9]. Patients with chronic immunodeficiency were excluded from the analysis when comparing the three groups based on glutamine levels in relation to lymphocyte count. Survival analyses were performed using Kaplan–Meier estimation (log-rank test). Bonferroni correction was applied for multiple comparisons. All *p*-values were two-sided, with significance set at <0.05. Statistical analysis was conducted using the R statistical computing environment (version 4.2.3) and SPSS (version 28.0.0).

## 3. Results

Out of 539 patients with suspected sepsis, 70 (13%) were identified by the data review committee as having acute illnesses that mimicked infection. Consequently, plasma amino acids were measured in 469 patients with sepsis. Their clinical characteristics and baseline plasma levels of the six amino acids are presented in Table 1.

The median SOFA score at IMU admission was 3 (interquartile range: 2–4). No patients presented with septic shock upon IMU admission. Mortality rates at 30 days and 6 months from IMU admission were 18% and 40%, respectively. In the logistic regression analysis (Table 2), the independent predictors of 6-month mortality included age (*p* < 0.001; OR 1.07 [95% CI: 1.05–1.10]), SOFA score (*p* < 0.001; OR 1.48 [95% CI: 1.32–1.66]), glutamine levels (*p* = 0.03; OR 1.77 [95% CI: 1.05–2.95]), and multiple sources of infection (*p* = 0.002; OR 3.54 [95% CI: 1.58–7.97]).

Patients with hypoglutaminemia (<400 μmol/L) comprised 47% (*n* = 219) of the cohort, while intermediate (400–700 μmol/L) and high glutamine (>700 μmol/L) levels were observed in 217 patients (46%) and 34 patients (7%), respectively (Table 3).

Subjects with hyperglutaminemia had a significantly higher median age compared to the other groups (*p* = 0.003). Lower respiratory tract infections and bloodstream infections were more common in patients with high (*p* = 0.013) and low (*p* = 0.035) glutamine levels, respectively.

At IMU admission, patients with glutamine levels above 700 μmol/L had significantly lower median body temperature, white blood cell count, lymphocyte count, and C-reactive protein levels compared to the other glutamine groups (*p* < 0.001, *p* = 0.008, *p* = 0.001, and *p* < 0.001, respectively; Table 3). Severe lymphopenia (lymphocytes < 0.5 × 10^9^/L) was more prevalent in hyperglutaminemic patients (32%) compared to their counterparts (*p* = 0.003). Admission hypoglutaminemia was associated with the lowest proportion of patients having lymphocyte counts < 0.5 × 10^9^/L at baseline (13%; *p* = 0.003).

The median SOFA score and lactate levels at baseline were significantly higher in patients with hyperglutaminemia (SOFA score: 4 (3–6); lactate: 17 mg/dL (13–26)) compared to those with hypoglutaminemia (SOFA score: 3 (2–4); *p* = 0.006, and lactate: 13 mg/dL (10–18); *p* = 0.004; Table 3). Patients with intermediate glutamine levels had a median SOFA score of 3 (2–4), which was not significantly different from that of hypoglutaminemic patients. However, median lactate concentrations were higher in the intermediate group (14 mg/dL (10–21); *p* = 0.004).

**Figure 1:**
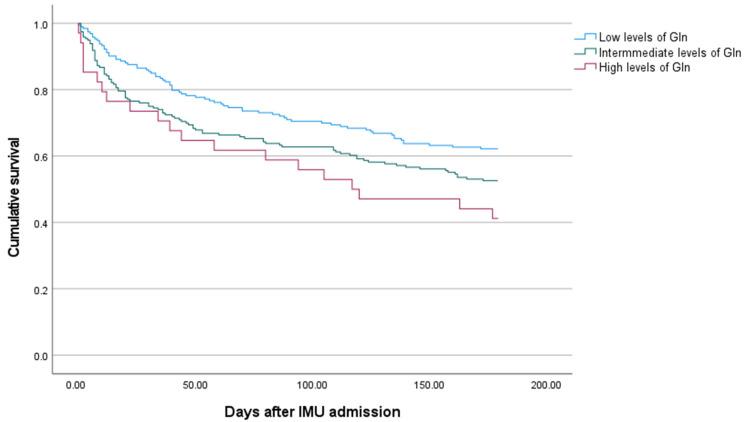
Kaplan–Meier curves of freedom from 6-month mortality for patients grouped according to glutamine levels. **ABBREVIATIONS:** Gln = glutamine and IMU = internal medicine unit. **LEGEND:** Low, intermediate, and high glutamine levels were defined as glutamine levels < 400 μmol/L, 400–700 μmol/L, and >700 μmol/L, respectively. Log-rank (30 days), *p* = 0.003; log rank (6 months), *p* = 0.005.

Significant differences in mortality were observed at 30 days (*p* = 0.016) and 6 months (*p* = 0.09) after IMU admission among patients with low (13% and 34%, respectively), intermediate (23% and 42%, respectively), and high (27% and 59%, respectively) glutamine levels. Kaplan–Meier analysis revealed an association between the three groups of septic patients based on glutamine levels and mortality at 30 days (log-rank *p* = 0.03) and 6 months (log-rank *p* = 0.05; Figure 1).

At baseline (Table 3), patients with low and intermediate glutamine levels showed significant differences in the concentrations of several amino acids: glutamic acid (171 μmol/L (116–225) vs. 128 μmol/L (97–184); *p* < 0.001), leucine (110 μmol/L (88–137) vs. 123 μmol/L (100–149); *p* = 0.009), and tyrosine (51 μmol/L (42–62) vs. 59 μmol/L (48–72); *p* < 0.001). They also differed in the ratios of glutamic acid/5-oxoproline (1.68 (1.41–1.86) vs. 1.41 (1.14–1.61); *p* < 0.001) and tyrosine/phenylalanine (0.90 (0.86–0.94) vs. 0.92 (0.88–0.96); *p* = 0.031).

Patients with intermediate glutamine levels had significantly lower median plasma concentrations of glutamic acid (128 μmol/L (97–184)), 5-oxoproline (101 μmol/L (78–129)), and tyrosine (59 μmol/L (48–72)) compared to hyperglutaminemic patients (156 μmol/L (130–218), *p* = 0.038; 139 μmol/L (117–176), *p* < 0.001; and 85 μmol/L (58–103), *p* < 0.001, respectively). The median tyrosine/phenylalanine ratio was also significantly lower in the intermediate group (0.92 (0.88–0.96)) than in the hyperglutaminemic group (0.95 (0.90–1.00); *p* = 0.014).

Compared to hypoglutaminemic patients, hyperglutaminemic individuals had higher median levels of 5-oxoproline (105 μmol/L (79–141) vs. 139 μmol/L (117–176); *p* < 0.001), phenylalanine (77 μmol/L (68–95) vs. 94 μmol/L (76–138); *p* = 0.003), and tyrosine (51 μmol/L (42–62) vs. 85 μmol/L (58–103); *p* < 0.001). Moreover, patients in the hyperglutaminemic group exhibited significantly higher glutamic acid/5-oxoproline (1.68 (1.41–1.86) vs. 1.19 (0.97–1.50)) and tyrosine/phenylalanine (0.95 (0.91–1.00) vs. 0.90 (0.86–0.94)) ratios compared to those with hypoglutaminemia (all *p* < 0.001).

## 4. Discussion

The main findings of this study are that patients with higher plasma glutamine levels exhibited a blunted inflammatory response and more pronounced lymphopenia at IMU admission. Moreover, their amino acid profiles showed alterations that indirectly suggest increased oxidative stress, glutathione depletion, anabolic resistance, and enhanced endogenous energy release. Overall, lymphopenia, specific metabolic derangements, and mortality rates progressively increased with rising glutamine levels—from low to intermediate to high.

The prevalence of each patient group based on glutamine levels varies significantly across studies [2,3,4,5,7,8,9] due to several factors. Our research focused exclusively on patients with sepsis, whereas previous studies included heterogeneous cohorts of critically ill individuals with a wide range of admission diagnoses [2,3,4,5,7,8,9]. Differences in the glutamine cut-off values used to define the various groups also contribute to this variability. In our study, hyperglutaminemia was defined as glutamine levels >700 μmol/L [4,9], whereas Sedberg et al. used a threshold of 930 μmol/L [8]. Based on their criteria, only 1% of our patients would be classified as hyperglutaminemic. The prevalence of organ dysfunction further influences glutamine levels [8]. In Sedberg’s study, 85% of patients had liver disease at admission, likely contributing to the higher frequency of glutamine levels exceeding 930 μmol/L. In contrast, only 6% of our patients had chronic liver disease, and none presented with acute liver failure at IMU admission. Our cohort primarily included older patients with less severe conditions than those typically admitted to ICUs, which may also explain the lower glutamine levels observed. Additionally, we found that sepsis originating from the lower respiratory tract was more prevalent among patients with hyperglutaminemia, reinforcing the previously suggested association between pulmonary sources of infection and elevated glutamine levels [22]. Finally, differences in infection characteristics and the exclusion of ‘do not resuscitate’ patients in some studies [8] may further account for the inconsistent distribution of low, intermediate, and high glutamine levels across different cohorts.

We explored potential pathophysiological mechanisms that might explain our findings, acknowledging that the interpretations presented are based on associative observations. In the context of sepsis, admission hyperglutaminemia may arise from several mechanisms, including excessive muscle catabolism and organ dysfunction [8,22]. The elevated lactate levels observed in patients with high plasma glutamine concentrations may support the former hypothesis. However, no significant differences were found in median levels of blood urea nitrogen, creatinine, aspartate aminotransferase, alanine aminotransferase, bilirubin, or INR among the three glutamine groups. Chronic liver disease was significantly more prevalent in patients with high glutamine levels, affecting 29% of this group, compared to those with lower concentrations. Patients with hyperglutaminemia also demonstrated more pronounced lymphopenia at baseline. As lymphocyte proliferation, differentiation, and activation are highly dependent on glutamine uptake [5], our findings may suggest either a severe impairment in these processes or significant dysfunction in immune cells’ ability to utilize glutamine. This supports the hypothesis that hyperglutaminemia is associated with a more immunosuppressed phenotype in sepsis, further corroborated by the lower median body temperature observed in this group at admission. Notably, lactate has been shown to exert immunosuppressive effects in preclinical studies; for instance, it promotes M2 polarization of macrophages through activation of the mammalian target of rapamycin (mTOR) pathway [23]. In addition to hyperlactatemia, immune suppression in sepsis is driven by metabolic reprogramming, including a shift from glycolysis to fatty acid oxidation, a compensatory response to mitochondrial dysfunction caused by excessive reactive oxygen species (ROS) [23]. At IMU admission, patients with hyperglutaminemia exhibited the lowest median glutamic acid to 5-oxoproline ratio, indirectly suggesting profound glutathione depletion [13,14,15] likely due to ROS overproduction and resultant redox imbalance [24,25]. Moreover, phenylalanine accumulation—possibly reflecting increased consumption of tetrahydrobiopterin, a key cofactor for phenylalanine hydroxylase—may also result from oxidative stress [16,26,27,28]. The elevated phenylalanine levels observed in hyperglutaminemic patients reinforce the likelihood of substantial oxidative stress in this group. These patients also had a higher tyrosine/phenylalanine ratio at admission. As tyrosine is a precursor for catecholamines such as dopamine, norepinephrine, and epinephrine [26,27,28], we hypothesize that enhanced production of stress hormones may shift this ratio toward tyrosine in individuals with elevated glutamine. This group also demonstrated more severe illness at baseline, as reflected by higher SOFA and lactate levels. Finally, elevated plasma leucine concentrations were observed in hyperglutaminemic patients. Leucine, a branched-chain amino acid, activates the mTOR pathway, which regulates autophagy, ribosomal biogenesis, tissue anabolism, and immune cell function [27,28,29,30]. The presence of elevated leucine may reflect significant anabolic resistance and increased endogenous energy release, possibly due to severe mitochondrial dysfunction and impaired beta-oxidation.

Patients with hypoglutaminemia exhibited higher clinical and laboratory markers of inflammation, along with less severe lymphocytopenia at baseline. The reduced levels of glutamine in these patients may be attributable to an increased demand for this amino acid by a larger number of active lymphocytes, which exceeds the body’s capacity to produce it [31,32]. The amino acid profile changes observed in hypoglutaminemic patients support the hypothesis that this condition is an epiphenomenon of a more robust immune response at baseline. Admission hypoglutaminemia was associated with a higher median glutamic acid to 5-oxoproline ratio, along with lower median levels of phenylalanine and leucine. These findings suggest that in patients with low glutamine levels, glutathione stores are relatively well preserved, ROS neutralization is more effective, and metabolic disruptions, such as anabolic resistance and endogenous energy release, are less pronounced. Moreover, patients with hypoglutaminemia had the highest concentrations of C-reactive protein at IMU admission. This may indicate an increased utilization of glutamine for synthesizing acute-phase proteins [4,5], further contributing to the low glutamine levels observed. Additionally, animal studies have shown that interleukin-1 and tumor necrosis factor-alpha can inhibit glutamine synthetase, thereby reducing endogenous glutamine production [33]. This mechanism may help explain the glutamine deficiency observed in our study, as higher median body temperatures at baseline were specifically associated with patients exhibiting hypoglutaminemia.

Patients with glutamine levels between 400 and 700 µmol/L exhibited more pronounced lymphopenia and metabolic disturbances compared to those with hypoglutaminemia, although these abnormalities were less severe than in patients with hyperglutaminemia. Specifically, patients with intermediate glutamine levels showed lower glutathione stores, and greater sepsis severity compared to those with hypoglutaminemia.

The relationship between low, intermediate, and high plasma glutamine levels and immune–inflammatory profiles, along with metabolic abnormalities observed in this study, may help explain the mortality rates at 30 days and 6 months from IMU admission. Compared to hypoglutaminemic patients, we hypothesize that individuals with higher glutamine levels may experience greater difficulty in eliminating the primary infection, placing them at increased risk for secondary infections and post-sepsis syndrome [10,11]. This, in turn, could contribute to the higher short- and long-term mortality rates observed in these patients.

This study has several notable strengths. It represents the largest cohort of internal medicine patients with sepsis in the literature, with measured baseline glutamine levels. The comprehensive evaluation of multiple clinical parameters and other amino acids has allowed us to propose potential mechanisms underlying the variation in glutamine levels among septic patients, as well as the poorer outcomes observed in those with elevated glutamine concentrations. These hypotheses may provide a foundation for future mechanistic and interventional studies targeting specific patient subgroups [34]. Older adults are often underrepresented in sepsis research [35,36,37,38,39], despite being the population most affected by severe infections. Our study specifically addresses elderly and very elderly patients with sepsis, a group at heightened risk for adverse outcomes. Finally, by ensuring that the study population closely reflects real-world patients, we offer insights that are particularly relevant to the clinical setting. This is essential for delivering valuable insights to physicians—particularly those in internal medicine—who are increasingly responsible for managing patients of advanced age, heightened vulnerability, and greater clinical complexity.

This research has several limitations. Elevated baseline glutamine levels were identified as an independent predictor of six-month mortality in internal medicine patients with sepsis. However, intervention studies are needed to determine whether glutamine could be useful in guiding early care escalation and monitoring sepsis patients during and after their stay [35,36,37,38,39] in the IMU. Furthermore, causal relationships between glutamine levels, immune suppression [40], and metabolic disturbances can only be speculated upon in this study. While lymphocyte count is a valuable marker of immune suppression [12], it may not fully capture the complexity of immune dysfunction in sepsis [11]. Additionally, lymphocyte count and amino acid levels were measured only at IMU admission. Longitudinal monitoring of these markers throughout the course of sepsis could enhance the identification of immunosuppressed patients and their specific metabolic abnormalities. Lastly, factors such as gut barrier dysfunction may affect glutamine levels, although their impact on plasma glutamine concentrations was not evaluated in this study.

## 5. Conclusions

Low, intermediate, and high baseline glutamine concentrations are associated with worsening lymphopenia, greater metabolic disturbances, and increased mortality in internal medicine patients with sepsis. Our findings underscore the importance of personalized approaches to metabolic and immune monitoring in sepsis, which could inform the design of future clinical trials.

## Figures and Tables

**Table 1:** Summary of baseline clinical characteristics and outcomes of the septic patients analyzed in this study.

Characteristics	*n* = 469 (100)
Female	222 (47)
Median age	82 (75–88)
Median Charlson Comorbidity Index	3 (2–5)
Chronic liver disease	30 (6)
Chronic immune deficiency	81 (17)
**Severity of sepsis at IMU admission**	
Median SOFA score	3 (2–4)
Median lactate (mg/dL)	14 (10–20)
**Amino acids at IMU admission**	
Median glutamine (μmol/L)	414 (324–518)
Median glutamic acid (μmol/L)	148 (104–214)
Median leucine (μmol/L)	117 (93–145)
Median 5-oxoproline (μmol/L)	106 (79–137)
Median phenylalanine (μmol/L)	81 (69–98)
Median tyrosine (μmol/L)	55 (46–70)
**Ratio of amino acids ^§^ at IMU admission**	
Median glutamic acid/5-oxoproline	1.52 (1.24–1.73)
Median tyrosine/phenylalanine	1.48 (1.23–1.77)
**Source of sepsis**	
Multiple sources of infection	37 (8)
LRTI	271 (63)
Non-LRTI	161 (37)
**Etiology of sepsis**	
Clinically documented	287 (61)
Microbiologically documented	182 (39)
-Gram-negative bacteria	120 (26)
-Gram-positive bacteria	83 (18)
-Non-bacterial	23 (5)
Positive blood cultures	86 (18)
**Mortality**	
<30 days	86 (18)
<6 months	186 (40)

**ABBREVIATIONS:** IMU = internal medicine unit, SOFA = sequential organ failure assessment, and LRTI = lower respiratory tract infection. **LEGEND:** Categorical data are reported as absolute frequencies and percentages, while continuous data are presented as medians with interquartile ranges. ^§^ expressed as log levels.

**Table 2:** Independent predictors of 6-month mortality: logistic regression analysis.

Predictor *	B	SE	Wald	*p*	Odds Ratio (95% CI)
Age	0.072	0.013	31.228	<0.001	1.07 (1.05–1.10)
SOFA score	0.391	0.059	43.83	<0.001	1.48 (1.32–1.66)
Log glutamine levels	0.569	0.263	4.498	0.030	1.77 (1.05–2.95)
Multiple sources of infection	1.262	0.413	9.358	0.002	3.54 (1.58–7.92)

**ABBREVIATIONS:** B = regression coefficient, SE = standard error, 95% CI = 95% confidence interval, and SOFA = sequential organ failure assessment. **LEGEND:** * covariates: gender, Charlson Comorbidity Index, lactate levels, tyrosine levels, and log glutamic acid levels/Log 5-oxo-proline levels.

**Table 3:** Comparison of patient characteristics and outcomes according to low (<400 μmol/L), intermediate (400–700 μmol/L), and high (>700 μmol/L) baseline glutamine levels: significant differences between the three groups.

Characteristics	Gln < 400 μmol/L *n* = 217 (46)	Gln 400–700 μmol/L *n* = 218 (47)	Gln > 700 μmol/L *n* = 34 (7)	*p*
Median age	80 (74–86) ^a^	83 (77–90)	84 (73–91)	0.003
Chronic liver disease	6 (4)	16 (10)	8 (29)	<0.001
Chronic immune deficiency	49 (23)	29 (13) ^b^	3 (9) ^c^	0.015
**Inflammation markers at IMU admission**				
Median body temperature (°C)	37.8 (36.8–38) ^a^	37.1 (36.1–38)	36.5 (36–37.5) ^c^	<0.001
Median white blood cell count (×10^9^/L)	13.7 (10.3–18.2) ^a^	12.3 (8.4–16.5)	11.5 (7.3–15-9)	0.008
Median C-reactive protein (mg/L)	138 (53–231) ^a^	90 (28–152) ^b^	33 (7–61) ^c^	<0.001
Lymphocyte count at IMU admission *				
Median lymphocyte (×10^9^/L)	1 (0.72–1.4) ^a^	0.78 (0.5–1.2)	0.72 (0.42–1.2)	0.001
Lymphocytes < 0.5 × 10^9^/L	21 (13) ^a^	47 (25)	10 (32) ^c^	0.003
**Severity of sepsis at IMU admission**				
Median SOFA score	3 (2–4)	3 (2–4) ^b^	4 (3–6) ^c^	0.006
Median lactate (mg/dL)	13 (10–18) ^a^	14 (10–21)	17 (13–26) ^c^	0.002
**Amino acids at IMU admission**				
Median glutamic acid (μmol/L)	171 (116–255) ^a^	128 (97–184) ^b^	156 (130–218)	<0.001
Median leucine (μmol/L)	110 (88–137) ^a^	123 (100–149)	123 (94–154)	0.009
Median 5-oxoproline (μmol/L)	105 (79–141)	101 (78–129) ^b^	139 (117–176) ^c^	<0.001
Median phenylalanine (μmol/L)	77 (68–95)	84 (70–99)	94 (76–138) ^c^	0.002
Median tyrosine (μmol/L)	51 (42–62) ^a^	59 (48–72) ^b^	85 (58–103) ^c^	<0.001
**Ratios between amino acids ^§^ at IMU admission**				
Median glutamic acid/5-oxoproline	1.68 (1.41–1.86) ^a^	1.41 (1.14–1.61)	1.19 (0.97–1.5) ^c^	<0.001
Median tyrosine/phenylalanine	0.90 (0.86–0.94) ^a^	0.92 (0.88–0.96) ^b^	0.95 (0.91–1) ^c^	<0.001
**Source of sepsis**				
LRTIs ^^^	109 (55)	141 (70) ^b^	21 (66) ^c^	0.013
**Etiology of sepsis**				
Positive blood cultures	46 (21)	38 (17) ^b^	2 (6) ^c^	0.035
**Mortality**				
<30 days	28 (13)	49 (23) ^b^	9 (27) ^c^	0.016
<6 months	73 (34)	93 (42) ^b^	20 (59) ^c^	0.009

**ABBREVIATIONS:** Gln = glutamine, IMU = internal medicine unit admission, SOFA = sequential organ failure assessment, and LRTIs = lower respiratory tract infections. **LEGEND:** Categorical data are reported as absolute frequencies and percentages, while continuous data are presented as medians with interquartile ranges. Bonferroni correction was applied for the comparison of characteristics among the three glutamine groups. * patients with chronic immunodeficiency were excluded from the analysis when comparing the three groups based on glutamine levels in relation to lymphocyte count. ^ patients with multiple sources of sepsis were excluded from the analysis when comparing the three groups based on glutamine levels in relation to single source of sepsis. ^§^ expressed as log levels. ^a^ significant difference between low and intermediate glutamine levels. ^b^ significant difference between intermediate and high glutamine levels. ^c^ significant difference between high and low glutamine levels.

## Data Availability

The datasets from this study are available from the corresponding author on request.

## Supplementary Materials

The following supporting information can be downloaded at https://www.mdpi.com/article/10.3390/jcm14103313/s1. The online version contains supplementary material. Supplementary File S1. Definitions adopted in the study. Process of adjudication of the nature of acute illness in patients with suspected sepsis.

## Abbreviations

ICU = Intensive Care Unit, ESPEN = European Society for Clinical Nutrition and Metabolism, ED = Emergency Department, IMU = internal medicine unit, SOFA = sequential organ failure assessment, INR = International Normalized Ratio, OR = Odds Ratio, 95% CI = 95% confidence interval, Gln = glutamine, LRTI = lower respiratory tract infection, B = regression coefficient, SE = standard error, mTOR = mammalian target of rapamycin, and ROS = reactive oxygen species.
