# Supplementary material for: Low, Intermediate, and High Glutamine Levels Are Progressively Associated with Increased Lymphopenia, a Diminished Inflammatory Response, and Higher Mortality in Internal Medicine Patients with Sepsis
Source: J Clin Med. 2025 May 9;14(10):3313. doi: 10.3390/jcm14103313 (PMC12112633; doi:10.3390/jcm14103313)
Supplement: Supplementary file 1 [file jcm-14-03313-s001.zip › jcm-3583038-supplementary.pdf]

SUPPLEMENTARY MATERIAL

Low, Intermediate, and High Glutamine Levels Are Progressively Associated with Increased Lymphopenia, a Diminished Inflammatory Response, and Higher Mortality in Internal Medicine Patients with Sepsis

Filippo Mearelli [1,*,†], Alessio Nunnari [1,*,†], Federica Chitti [1], Annalisa Rombini [1], Alessandra Macor [1], Donatella Denora [1], Luca Messana [1], Marianna Scardino [1], Ilaria Martini [1], Giulia Bolzan [1], Noemi Merlo [1], Fabio Di Paola [1], Francesca Spagnol [1], Chiara Casarsa [1], Nicola Fiotti [1], Venera Costantino [2], Verena Zerbato [3], Stefano Di Bella [3], Carlo Tascini [4], Daniele Orso [5], Filippo Giorgio Di Girolamo [1] and Gianni Biolo [1]

1 Unit of Internal Medicine, Clinica Medica, Department of Medical Surgical and Health Sciences, University of Trieste, Strada di Fiume 447, 34100 Trieste, Italy
2 Microbiology Unit, University Hospital (ASUGI), Strada di Fiume n° 447, 34137 Trieste, Italy; venera.costantino@asugi.sanita.fvg.it
3 Infectious Diseases Unit, Clinical Departement of Medical, Surgical, and Health Sciences, University Hospital (ASUGI), Piazzale dell'Ospedale n° 1, 34129 Trieste, Italy; stefano932@gmail.com (S.D.B.)
4 Infectious Diseases Unit, University Hospital (ASUFC), Via Pozzuolo n° 330, 33100 Udine, Italy; c.tascini@gmail.com
5 Department of Anesthesia and Intensive Care, University Hospital (ASUFC), Via Pozzuolo n° 330, 33100 Udine, Italy; sd7782.do@gmail.com
* Correspondence: filippome@libero.it (F.M.); alessionunnari@gmail.com (A.N.); Tel.: +39-040-399-4762 (F.M.); +39-040-399-4762 (A.N.)
† These authors contributed equally to this manuscript.

TABLE OF CONTENTS

Additional file 1. Definitions adopted in the study. Process of adjudication of the nature of acute illness in patients with suspected sepsis.

DEFINITIONS ADOPTED IN THE STUDY

Severity

Septic shock: patients with SOFA ≥2 who, despite adequate fluid resuscitation, require vasopressors to maintain a mean arterial pressure ≥65 mmHg and have a lactate >18 mg/dL.

Etiology

Patients with definitive diagnosis of sepsis were further subdivided in clinically documented infections and microbiologically documented infections. Clinically documented infections comprised sepsis cases without supporting gram stain or cultures. Microbiologically documented infections included sepsis cases with microbiological confirmation of infection. Microbiologically documented infections were further subdivided into bacterial and nonbacterial infections depending on the type of germ cultured in sterile biological fluids or identified by ancillary diagnostic examinations.

Bacterial sepsis: sepsis in which causative pathogens were bacteria.
Polymicrobial sepsis: sepsis due to more than one bacterial pathogen.
Monomicrobial sepsis: sepsis due to one bacterial pathogen.
Non-bacterial sepsis: sepsis in which the pathogens of infection were viruses, protozoa or fungi.

Blood stream infection was defined as the presence of viable pathogen in the bloodstream. In this study, a single positive blood culture for organisms typically associated with skin flora (such as coagulase-negative staphylococci, corynebacterium, and alpha-hemolytic streptococci) was considered a contaminant.

Source of sepsis

Single source: all the single sources of infection different from respiratory tract (respiratory sepsis) were grouped in non-respiratory sepsis.
Multiple source: sepsis in which more than one site of infection were clinically and/or microbiologically documented

PROCESS OF ADJUDICATION OF THE NATURE OF ACUTE ILNESS IN PATIENTS WITH SUSPECTED SEPSIS

Data review committee was composed by Filippo Mearelli (internal medicine), Carlo Tascini (infectious diseases), and Venera Costantino (clinical microbiology). At the end of clinical follow-up (discharge or death of the patients from hospital), data review committee reviewed case reports forms and clinical charts of the patients with suspected sepsis. Nature of the acute process (sepsis vs sepsis mimickers), the etiology and characteristics of infections (bacterial vs non-bacterial; if bacterial: mono vs polymicrobial and bacteremic vs non-bacteremic; if monomicrobial: gram + vs gram -) and the source of infection (multiple vs single source; if single: respiratory vs non-respiratory) in patients with suspected sepsis admitted to internal medicine unit. An agreement on sepsis diagnosis was reached in all cases by multiple rounds of adjudication.
